# Morphine overinfusion of intrathecal drug administration system under magnetic resonance evaluation for the diagnosis of stroke: a case report

**DOI:** 10.1186/s12883-021-02176-x

**Published:** 2021-04-05

**Authors:** Seoyeon Kim, Min Kyoung Kang

**Affiliations:** 1 Department of Neurology, Seoul National University Hospital, Seoul, Republic of Korea; 2 Department of Neurology, Uijeongbu Eulji Medical Center, 712, Dongil-ro, Uijeongbu, 11759 Republic of Korea

**Keywords:** Intrathecal drug administration system, Morphine overdose, Magnetic resonance imaging, Case report

## Abstract

**Background:**

Until recently, it is generally considered safe to perform magnetic resonance imaging (MRI) in patients with an intrathecal drug administration system (ITDAS) device. In this study, we presented a case of morphine overdose due to ITDAS malfunction during MRI evaluation for the diagnosis of stroke.

**Case presentation:**

A 58-year-old woman was referred to the emergency department for left-sided hemiparesis and dysarthria. She had undergone ITDAS implantation 4 years ago because of intractable back pain. Her brain MRI examination did not show any abnormalities except an old hemorrhagic infarction in the right basal ganglia. After MRI was performed, her symptoms completely resolved. Approximately 3 h after the MRI scan, the patient showed progressive stuporous consciousness and decreased respiration with decreased peripheral oxygen saturation of 80%. Initial arterial blood gas analysis revealed respiratory acidosis with hypoxia and hypercapnia. We suspected the opioid overdose for her unconciousness, small and sluggish pupils, and slow respiration. The patient regained consciousness within 3 min after the administration of naloxone with severe anxiety and irritability, without any respiratory symptoms or focal neurological deficits. In the pump interrogation and actual reservoir checks performed 6 h after the MRI scan, there was no significant difference between the expected reservoir volume and actual reservoir volume. Follow-up MRI performed to rule out posterior circulation infarction showed no structural lesions. The patient was eventually discharged without further neurologic or functional deterioration, with diagnosis of transient ischemia attack for initial symptoms of focal neurologic deficits.

**Conclusion:**

Although both ex vivo and in vivo studies have provided evidence that ITDAS devices are MRI-compatible, the pump is made of titanium and has ferromagnetic components. Since misdiagnosis of overinfusion could lead to mortality, early awareness of overinfusion of the intrathecal drug is needed to all clinicians in case of performing MRI in ITDAS implanted patients.

## Background

After its first introduction in 1981, the intrathecal drug administration system (ITDAS) has been widely used to relieve intractable pain [1]. It is generally considered safe to perform magnetic resonance imaging (MRI) in patients with an ITDAS device [2]. As the use of ITDAS increased, attention is also been drawn to the risk of abrupt withdrawal or side effects of overinfusion due to the malfunction of ITDAS during MRI [2]. There are few reports on the stall of the pump motor of ITDAS during MRI. However, there are no reports on overinfusion due to the malfunction of ITDAS under MRI. In this study, we presented a case of morphine overdose due to ITDAS malfunction during MRI evaluation for the diagnosis of stroke.

## Case presentation

**Fig. 1:**
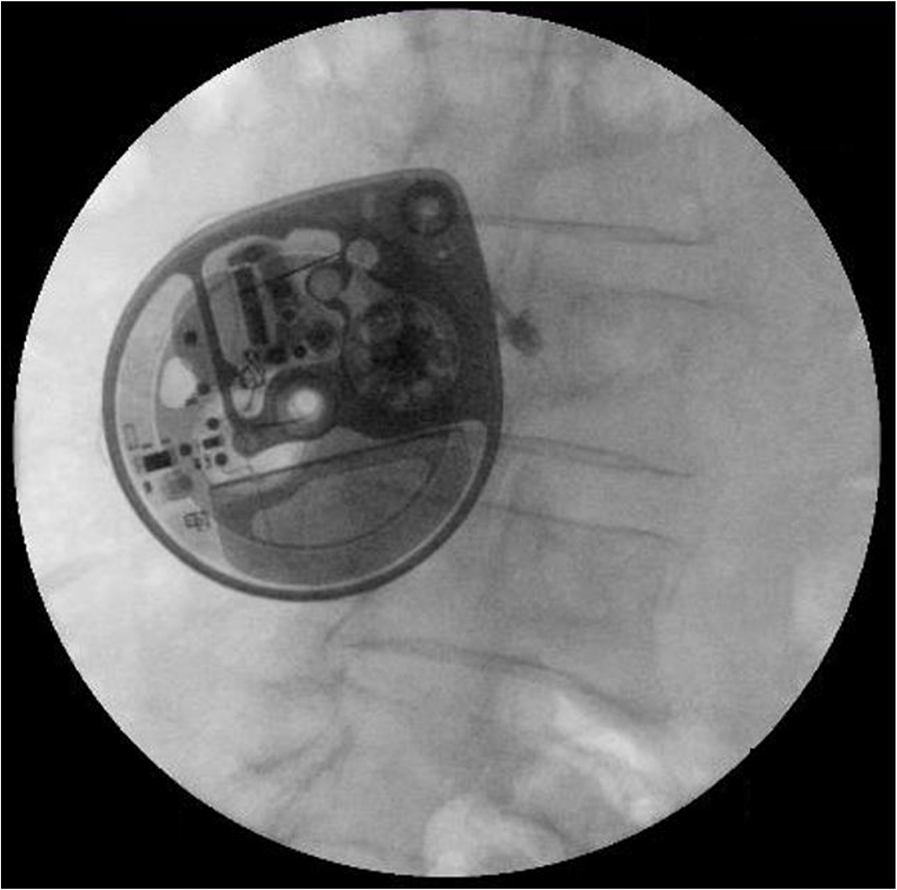
Intrathecal drug administration system examined by a fluoroscope

**Fig. 2:**
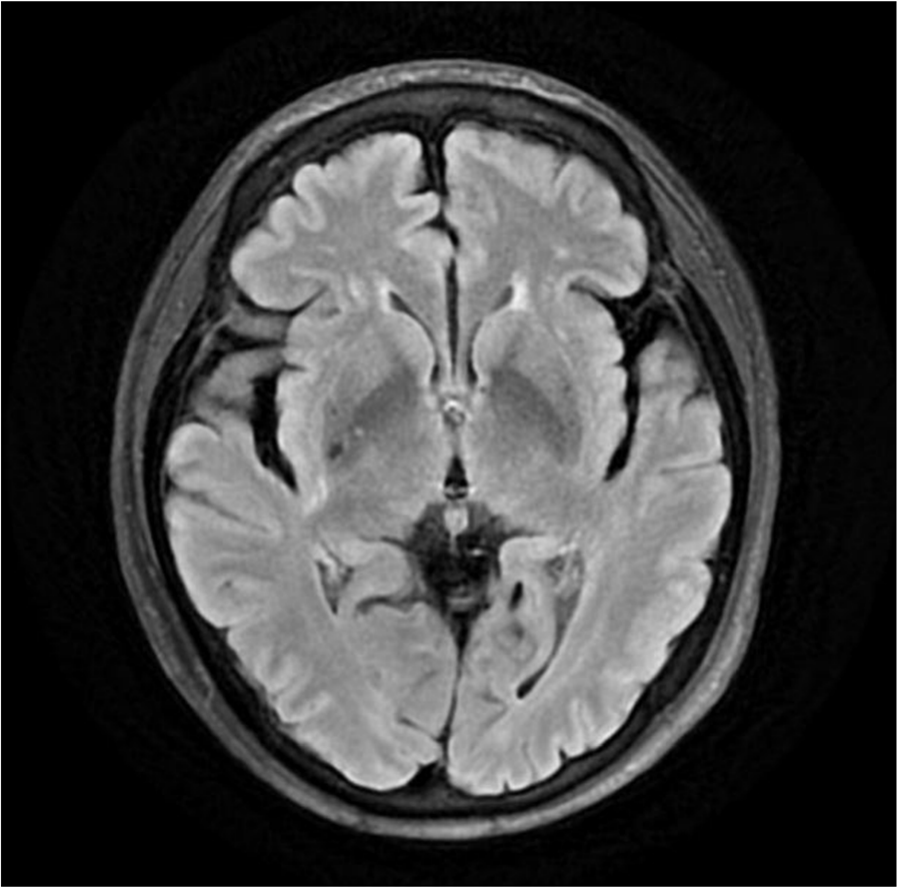
T2 magnetic resonance imaging showing old hemorrhagic infarct in the right basal ganglia

A 58-year-old woman was referred to the emergency department for left-sided hemiparesis and dysarthria which had occurred 6 h ago. Under neurologic examination, she showed grade IV weakness on manual muscle testing and 80% of tactile hypoesthesia of her left arm and leg. She had undergone ITDAS implantation 4 years ago. (SynchroMed^Ⓡ^, Medtronic, Minneapolis, MN) She was treated with intrathecal morphine at the anesthesiology department due to chronic intractable back pain that occurred after an L4–5 decompressive laminectomy 11 years ago, as shown in Fig. 1 (morphine concentration: 10 mg/ml, basal rate: 0.1653 mg/hr., Flex rate (8 h): 0.143 mg/hr., dose/day: 3.785 mg/day). 1.5 T MRI over 15 min at 0.2 W/Kg specific absorption rate performed after the initial examination did not show any abnormalities on diffusion-weighted MRI. An old hemorrhagic infarction in the right basal ganglia was seen on the T2 image (Fig. 2). After MRI was performed, her symptoms completely resolved. A transient ischemia attack for the current presentation was suspected, and the patient was admitted to the stroke unit for close monitoring.

Approximately 3 h after the MRI scan, the patient showed progressive stuporous consciousness and decreased respiration (8 breaths per minute) with decreased peripheral oxygen saturation of 80%. Her pupil measured 2 mm/2 mm and displayed a sluggish response to light. Initial arterial blood gas analysis revealed respiratory acidosis with hypoxia and hypercapnia (pH 7.280, pO2 51.8 mmHg, pCO2 69.9 mmHg). Manual resuscitator bagging with full oxygenation was performed. We suspected the opioid overdose for her unconsciousness, small and sluggish pupils, and slow respiration. The patient regained consciousness within 3 min after administration of the first dose of 0.4 mg IV of naloxone hydrochloride followed by repeated dose of 0.4 mg at 2 min interval, with severe anxiety and irritability. Subsequent respiratory symptoms or focal neurological deficits was not observed after the administration of the second dose of naloxone.

Pump interrogation and actual reservoir checks were performed 6 h after the MRI scan. There was no significant difference between the expected reservoir volume and actual reservoir volume. (expected reservoir volume: 11.2 cc, actual reservoir volume: 11.0 cc) Follow-up MRI performed to rule out posterior circulation infarction showed no structural lesions. The patient was eventually discharged without further neurologic or functional deterioration, with diagnosis of transient ischemia attack for initial symptoms of focal neurologic deficits.

## Discussion and conclusions

Although both ex vivo and in vivo studies have provided evidence that ITDAS devices are MRI-compatible, the pump is made of titanium and has ferromagnetic components, including a magnetic switch that poses a risk of malfunction in MRI settings. Magnetic fields can temporarily stop ITDAS pumps and suspend drug infusion for the entire duration of the MRI exposure, which can lead to the development of withdrawal symptoms, as reported in a previous case report [3]. However, the urgent life-threatening complication, morphine overdose is theoretically possible but has not yet been reported.

In this case, the pump could not be emptied or checked before the MRI exam because of hyperacute stroke condition. The main limitation of our case study is that the interrogation test revealed no difference between the expected reservoir volume and actual reservoir volume in ITDAS. We suspected that rotor of ITDAS was temporarily stalled before over-function or that there was a measurement error in interrogation test, but these two hypothesis could not be adequately supported as no previous studies have reported time series studies focusing on the function of ITDAS under MRI exposure or measurement variability of interrogation test. In addition, the neurologic examination during the event did not show classic pinpoint constricted pupils, and we cannot exclude the possibility of short-duration transient ischemia attack involving multiple cerebral territory. However, given the respiratory depression with stuporous mental status, a favorable response to naloxone, and no structural lesion detected by follow-up MRI, morphine intoxication caused by transient ITDAS over-function seems to be the most reasonable explanation. Drugs used in ITDAS, such as morphine, hydromorphone, bupivacaine, clonidine, baclofen, fentanyl, and sufentanil have adverse effects of overdose which includes; Somnolence, coma, hyporeflexia, respiratory depression, and cardiac conduction abnormalities [4]. Since misdiagnosis could lead to mortality, early awareness of overinfusion of the intrathecal drug is needed to all clinicians in case of performing MRI in ITDAS implanted patients.

## Data Availability

The data during the current study are available from the corresponding author on reasonable request.

## Abbreviations

MRI: Magnetic resonance imaging
ITDAS: Intrathecal drug administration system
